# Preparation of Self-Assembled Chitin Nanofiber-Natural Rubber Composite Sheets and Porous Materials

**DOI:** 10.3390/biom7030047

**Published:** 2017-07-01

**Authors:** Akito Kawano, Kazuya Yamamoto, Jun-ichi Kadokawa

**Affiliations:** Department of Chemistry, Biotechnology and Chemical Engineering, Graduate School of Science and Engineering, Kagoshima University, 1-21-40 Korimoto, Kagoshima 860-0065, Japan; k7046787@kadai.jp (A.K.); yamamoto@eng.kagoshima-u.ac.jp (K.Y.)

**Keywords:** chitin nanofiber, composite, natural rubber, porous material

## Abstract

We previously reported the preparation of a self-assembled chitin nanofiber (CNF) film via regeneration from an ion gel with an ionic liquid, followed by sonication and filtration. Based on the finding that CNFs were redispersed in a mixture of the film with ammonia aqueous solution (aq.), in this study, CNF-natural rubber (NR) composite sheets were fabricated by mixing redispersed CNF with NR latex stabilized by ammonia, followed by drying under reduced pressure. Tensile testing of the sheets indicated the reinforcing effect of CNFs. Further, CNF-NR composite porous materials were fabricated by evaporating ammonia from the CNF-NR dispersion, followed by lyophilization. The mechanism for the formation of porous structures was evaluated.

## 1. Introduction

**Figure 1:**
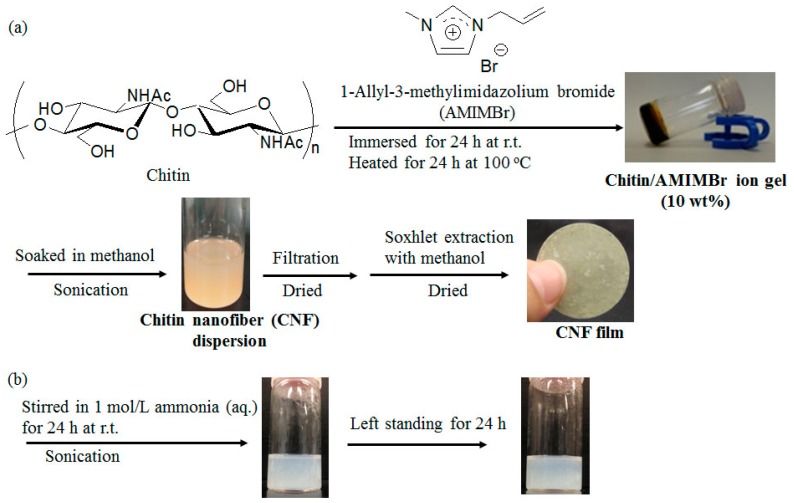
(**a**) Preparation of self-assembled CNF dispersion and film through regeneration from ion gel with AMIMBr using methanol; and (**b**) redispersion of CNF film in 1 mol/L ammonia aqueous solution (aq.).

Biopolymers are widely distributed and exhibit specific vital functions in nature [1,2]. Chitin and natural rubber (NR, *cis*-1,4-polyisoprene) are representative biopolymers with highly crystalline and amorphous natures, respectively [3,4,5,6]. Chitin is an aminopolysaccharide composed of β(1→4)-linked *N*-acetyl-d-glucosamine repeating units and is present in the exoskeletons of crustaceans, shellfish, and insects. It still remains an unutilized biomass resource, primarily because of its intractable bulk structure and insolubility in water and common organic solvents. Therefore, research concerning the conversion of chitin to various useful bio-based materials after proper dissolution in suitable solvents has attracted much attention in recent years [7]. Some ionic liquids such as imidazolium acetates with different substituents were found to dissolve chitin [8,9,10]. We also reported that an ionic liquid, 1-allyl-3-methylimidazolium bromide (AMIMBr), dissolves chitin in concentrations up to 4.8 weight percent (wt%) and further forms ion gels with higher contents of chitin [11,12]. We found in a subsequent publication that chitin self-assembles to form nanofiber dispersions by regeneration from the ion gel using methanol (Figure 1a). When the dispersion was filtered, the isolated self-assembled chitin nanofibers (CNFs) got highly entangled resulting in the formation of a film [13,14,15]. The dispersion has been used with other polymeric components such as cellulose, carboxymethyl cellulose, and poly(vinyl alcohol) to obtain CNF-containing composite materials [13,16,17]. As nanofibrillation is one of the efficient approaches for the functionalization of chitin, CNFs prepared by various techniques have been employed to fabricate composite materials besides those in the above studies [7,13,14,18,19,20,21].

For example, CNFs prepared by acid-hydrolysis were used as reinforcing agents for NR [22,23,24]. NR is obtained from plants as NR latex, in which *cis*-1,4-polyisoprene particles are dispersed in an aqueous continuous phase. NR forms a stable colloid in basic aqueous solutions, such as ammonia solution, because the particle surface is negatively charged [25]. NR is one of the most important elastomers used in industry and it has been found that the properties of NR can be tailored by the addition of fillers of varying aggregate size and aspect ratio [26]. For additional functionalization of NR, porous films were fabricated loaded with gentamicin sulfate for wound dressings [27].

**Figure 2:**
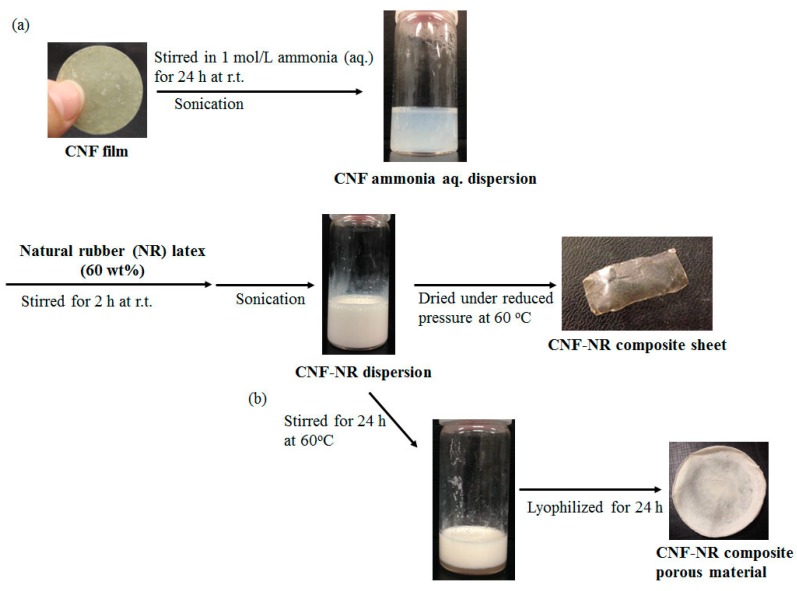
(**a**) Preparation of self-assembled CNF-NR composite sheet; and (**b**) self-assembled CNF-NR composite porous material.

Based on the finding that the aforementioned self-assembled CNFs are redispersed in aqueous media under weakly basic conditions, such as in ammonia aqueous solution (aq.) (Figure 1b), in this study we investigated the preparation of CNF-NR composite sheets via mixing the CNF-ammonia (aq.) dispersion with NR latex stabilized with ammonia, followed by drying (Figure 2a). Further, composite porous materials were also fabricated through the formation of CNF-NR aggregates by the evaporation of ammonia stabilizer from the mixture (Figure 2b).

## 2. Results and Discussion

### 2.1. Preparation of Self-Assembled CNF-NR Composite Sheets

**Figure 3:**
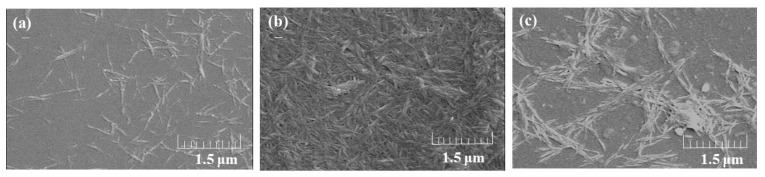
Scanning electron microscopy (SEM) images of (**a**) dispersion in methanol; (**b**) film; and (**c**) redispersion in 1 mol/L ammonia aq. of self-assembled CNFs.

As previously reported [13], the self-assembled CNF dispersion with methanol was prepared via gelation with AMIMBr followed by regeneration using methanol. The dispersion was then filtered to isolate CNFs and produce the self-assembled CNF film (Figure 1a). When the film was immersed in 1 mol/L ammonia (aq.) for 24 h at room temperature with stirring followed by sonication for 1 h, the film was disentangled and the CNFs were gradually dispersed, probably owing to weakening hydrogen bonds under such weakly basic conditions (Figure 1b). The dispersed state was stable after standing for 24 h, indicating good redispersion of CNFs in ammonia (aq.). Scanning electron microscopy (SEM) image of the resulting dispersion (Figure 3c) shows the relatively independent nanofiber morphology, which is different from that of the CNF film (Figure 3b). Although the aggregated morphology of CNFs after redispersion is also seen (compared with the SEM image of the original dispersion obtained by regeneration from the ion gel using methanol (Figure 3a)), the SEM result suggests the redispersible property of CNFs in ammonia (aq.).

**Figure 4:**
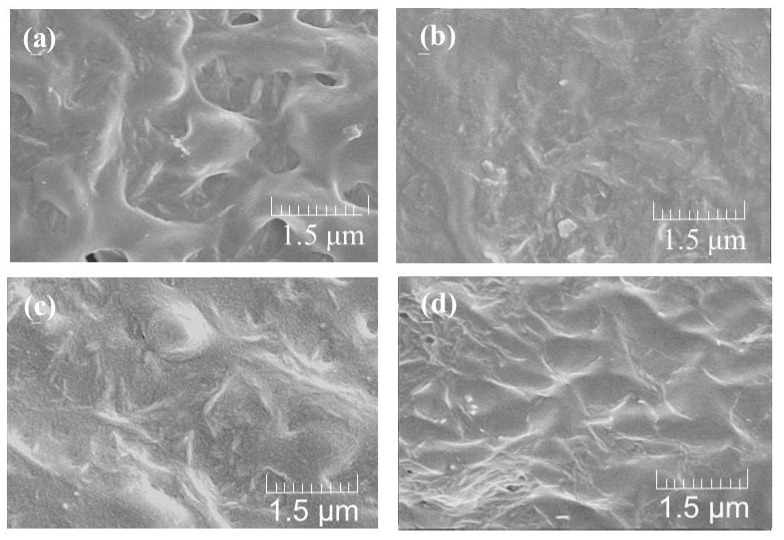
SEM images of self-assembled CNF-NR composite sheets (**a**–**d**;CNF:NR (weight ratio) = 0.01, 0.02, 0.1, 0.2:1, respectively.

**Figure 5:**
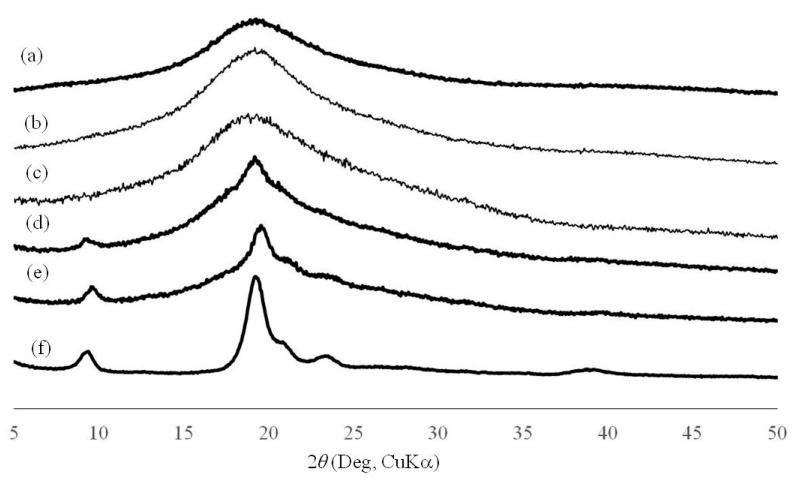
X-ray diffraction (XRD) profiles of (**a**) NR; (**b**–**e**) self-assembled CNF-NR composite sheets (CNF:NR (weight ratio) = 0.01, 0.02, 0.1, 0.2:1, respectively); and (**f**) self-assembled CNF film.

**Figure 6:**
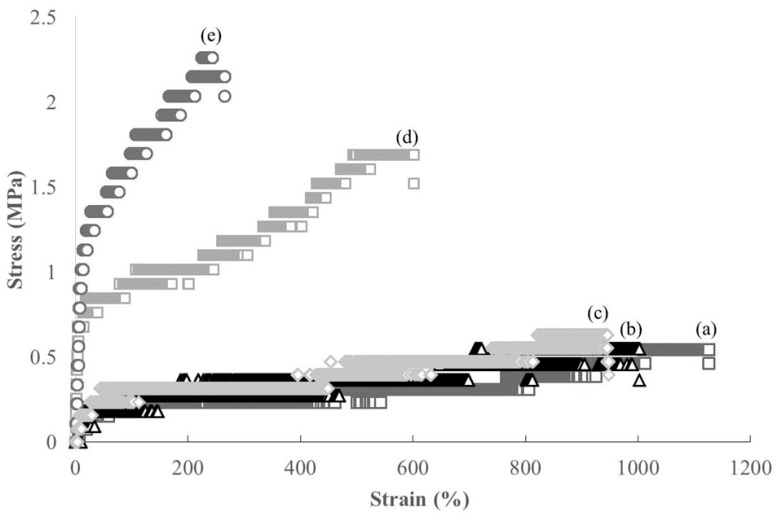
Stress–strain curves of (**a**) NR; and (**b**–**e**) self-assembled CNF-NR composite sheets (CNF:NR (weight ratio) = 0.01, 0.02, 0.1, 0.2:1, respectively) under tensile mode.

The redispersion of CNFs with ammonia (aq.) was mixed with NR latex (60 wt%) to fabricate CNF-NR composite materials at different CNF weight ratios (CNF:NR = 0.01, 0.02, 0.1, 0.2:1), because latex is stabilized with ammonia (Figure 2a). After the mixture was stirred for 2 h at room temperature, it was sonicated for 1 h to give a dispersion of NR with CNF. The dispersion was dried under reduced pressure for 4 h at 60 °C in a drying tray to obtain a CNF-NR composite sheet. SEM images of the resulting sheets with different weight ratios show the relatively independent nanofiber morphologies in areas of NR solids (Figure 4). These results suggest that CNFs were dispersed well in the NR latex. Further, with the increasing weight ratio of CNFs, the nanofiber morphologies are more clearly seen in the SEM images of the composite sheets. X-ray diffraction (XRD) profiles of the composite sheets with lower CNF weight ratios (CNF:NR = 0.01, 0.02:1) did not show obvious crystalline peaks, similar to that of a pure NR sheet (Figure 5a–c) that was prepared by drying NR latex. With increasing weight ratios of CNFs (CNF:NR = 0.1, 0.2:1), the diffraction peaks assigned to the crystal structure of chitin were gradually detected (Figure 5d–f). These results indicate the retention of the crystal structure of chitin in the composite. The mechanical properties of the composite sheets were evaluated by tensile testing. The resulting stress–strain curves exhibited that with increasing weight ratio of CNF, the tensile strength and Young’s modulus increased, whereas the elongation values at break decreased (Figure 6 and Table 1). Particularly, these values for the compositions with higher weight ratios of CNFs (CNF:NR = 0.1, 0.2:1), were significantly different from those of a pure NR sheet. These results support the effects of strengthening of mechanical properties and the reinforcement with increasing weight ratio of CNFs.

### 2.2. Preparation of Self-Assembled CNF-NR Composite Porous Materials

**Figure 7:**
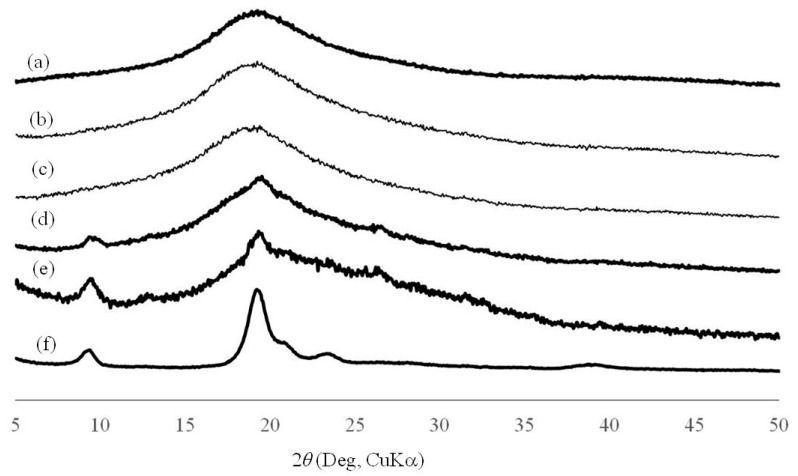
XRD profiles of (**a**) NR; (**b**–**e**) self-assembled CNF-NR composite porous materials (CNF:NR (weight ratio) = 0.01, 0.02, 0.1, 0.2:1, respectively); and (**f**) self-assembled CNF film.

**Figure 8:**
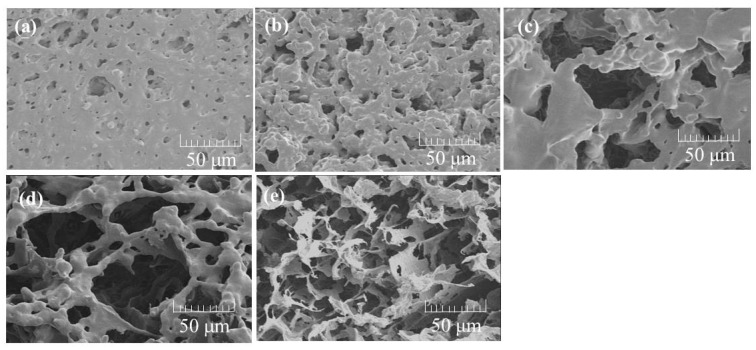
SEM images of (**a**) NR sheet; and (**b**–**e**) self-assembled CNF-NR composite porous materials (CNF:NR (weight ratio) = 0.01, 0.02, 0.1, 0.2:1, respectively).

The CNF-NR porous materials were fabricated by different preparation procedures from those above (Figure 2b). The CNF-NR dispersions with different weight ratios (CNF:NR = 0.01, 0.02, 0.1, 0.2:1), which were prepared by the same procedure as above (Figure 2a), were heated for 24 h at 60 °C with stirring to evaporate the ammonia stabilizer. The resulting mixtures were then lyophilized to prepare the composite materials. The XRD profiles of the composites show similar results to those of the above composite sheets in which the diffraction peaks assigned to the crystal structure of chitin gradually increased with increasing weight ratio of CNF (Figure 7). The SEM image of the composite with lower CNF weight ratio (CNF:NR = 0.01:1) shows a morphology with a few small pores (Figure 8b), which is different from that of a pure NR sheet (Figure 8a). With increasing weight ratio of CNF (CNF:NR = 0.02, 0.1:1), the SEM images showed increases in the number and size of pores, indicating the formation of porous materials (Figure 8c,d). With further increasing weight ratio of CNF (CNF:NR = 0.2:1), however, obvious frameworks for the porous morphology were not seen in the SEM image (Figure 8e). Indeed, the material showed a brittle nature.

**Figure 9:**
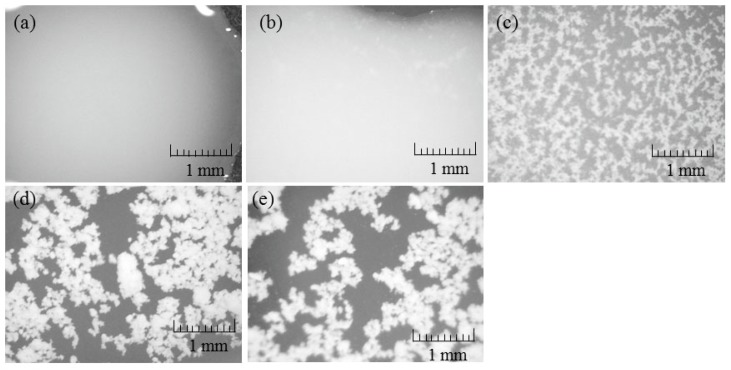
Charge coupled device images of (**a**) NR latex; and (**b**–**e**) self-assembled CNF-NR dispersions (CNF:NR (weight ratio) = 0.01, 0.02, 0.1, 0.2:1, respectively) after evaporation of ammonia.

**Figure 10:**
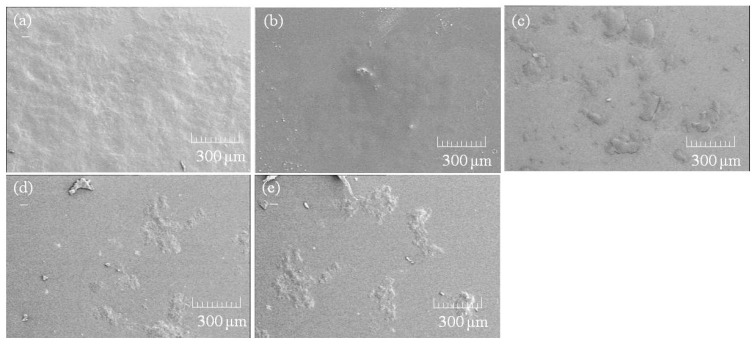
SEM images of (**a**) NR latex; and (**b**–**e**) self-assembled CNF-NR dispersions (CNF:NR (weight ratio) = 0.01, 0.02, 0.1, 0.2:1, respectively) after evaporation of ammonia.

To evaluate the mechanism for the formation of the porous structure, a charge coupled device (CCD) camera view of the CNF-NR mixtures after the evaporation of ammonia was recorded (Figure 9). CCD images show the formation of larger aggregates with an increasing weight ratio of CNF, whereas such aggregates are not seen in the image of the NR latex. SEM images of the same samples also observed the aggregate morphologies (Figure 10). These results suggest that CNFs aggregate with NR in the absence of ammonia stabilizer. Lyophilization of the mixtures caused agglomeration of the aggregates with the formation of spaces between them to construct the porous structure. The presence of larger amounts of CNFs provided larger aggregates, resulting in the increased number and size of pores, leading to unclear frameworks with large spaces.

## 3. Materials and Methods

### 3.1. Materials

Chitin powder from crab shell was purchased from Wako Pure Chemicals, Tokyo, Japan. NR latex (60 wt%, Selatex 1100) was supplied by Sumitomo Rubber Industries, Ltd., Kobe, Hyogo, Japan. Ionic liquid AMIMBr was prepared by the reaction of 1-methylimidazole with 3-bromo-1-propene according to a method adapted from the literature [28]. Other reagents and solvents were available commercially and used without further purification.

### 3.2. Preparation of Self-Assembled CNF Film

A mixture of chitin (0.120 g, 0.59 mmol) with AMIMBr (1.00 g, 4.92 mmol) was allowed to stand at room temperature for 24 h and subsequently heated with stirring at 100 °C for 24 h to obtain a chitin ion gel (10 wt%). The gel was then soaked in methanol (40 mL) at room temperature for 48 h, followed by sonication (Branson 1510, Branson Ultrasonics, Emerson Japan, Ltd., Atsugi, Japan (42 kHz, 70 W)) for 10 min to yield a self-assembled CNF dispersion with methanol. The dispersion was subjected to filtration to isolate CNFs, which were dried for 3 h at 60 °C under reduced pressure to obtain a CNF film.

### 3.3. Redispersion of CNF Film in Aqueous Ammonia Solution

A mixture of CNF film (20.5 mg) with 1 mol/L aqueous ammonia (15 mL) was stirred for 24 h at room temperature, followed by sonication for 1 h. The resulting mixture was left standing for a further 24 h to evaluate redispersibility.

### 3.4. Preparation of Self-Assembled CNF-NR Composite Sheet

A typical experimental procedure was as follows (Run 1, Table 1). A mixture of the CNF film (10.4 mg) with 1 mol/L aqueous ammonia (15 mL) was stirred for 24 h at room temperature, followed by sonication for 1 h to redisperse. After the NR latex (60 wt%, 1.66 g) was mixed with the resulting dispersion, the mixture was stirred for 2 h at room temperature, followed by sonication for 1 h to yield the CNF-NR dispersion. The resulting dispersion was dried in a drying tray for 4 h at 60 °C under reduced pressure to obtain the CNF-NR composite sheet (0.918 g).

### 3.5. Preparation of Self-Assembled CNF-NR Composite Porous Material

A typical experimental procedure was as follows (Run 5, Table 1). A mixture of the CNF film (3.6 mg) with 1 mol/L aqueous ammonia (15 mL) was stirred for 24 h at room temperature, followed by sonication for 1 h to redisperse. After the NR latex (60 wt%, 0.509 g) was mixed with the resulting dispersion, the mixture was stirred for 2 h at room temperature, followed by sonication for 1 h to yield the CNF-NR dispersion. After the resulting dispersion was heated for 24 h at 60 °C with stirring, the mixture was lyophilized for 24 h to obtain the CNF-NR composite porous material (0.220 g).

### 3.6. Measurements

XRD measurements were conducted using a PANalytical X’Pert Pro MPD (PANalytical B.V., Almelo, The Netherlands) with Ni-filtered Cu-K_α_ radiation (*λ* = 0.15418 nm). SEM images were obtained using a Hitachi S-4100H electron microscope (Hitachi High-Technologies Corporation, Tokyo, Japan). Stress–strain curves were measured using a tensile tester (Little Senstar LSC-1/30, Tokyo Testing Machine, Tokyo, Japan). CCD camera observation was conducted using Dino-Lite Digital Microscope AM-311 (AnMo Electronics Corporation, Hsinchu, Taiwan).

## 4. Conclusions

In this paper, we reported the preparation of self-assembled CNF-NR composite sheets and porous materials. The self-assembled CNFs were redispersed by mixing the CNF film with ammonia (aq.) Accordingly, the CNF dispersion with ammonia (aq.) was mixed with NR latex stabilized with ammonia, followed by drying under reduced pressure to obtain CNF-NR composite sheets. SEM and XRD measurements were performed to evaluate the composition and crystal structure in the sheets, respectively. The tensile testing of the sheets confirmed the reinforcing effect of CNFs in the sheets. When the CNF-NR dispersion was heated to evaporate ammonia, porous materials were fabricated following lyophilization. By evaporating ammonia stabilizer, CNFs were aggregated with NR. The aggregates were then agglomerated, forming spaces between them to construct the porous structure.

## Figures and Tables

**Table 1:** Mechanical properties of CNF-NR composite sheets under tensile mode.

CNF:NR (wt Ratio)	Tensile Strength (MPa)	Elongation at Break (%)	Young’s Modulus (MPa)
NR	0.5	1125	0.4
0.01:1	0.5	1002	0.5
0.02:1	0.6	945	0.8
0.1:1	1.7	602	8.8
0.2:1	2.3	247	10.7
